# Predicting compressive and splitting tensile strength of high volume fly ash roller compacted concrete using ANN and ANN-biogeography based optimization models

**DOI:** 10.1038/s41598-025-05700-y

**Published:** 2025-07-01

**Authors:** Murteda Unverdi, Ramin Kazemi, Yahya Kaya, Naz Mardani, Ali Mardani, Seyedali Mirjalili

**Affiliations:** 1 Civil Engineering Department, Bursa Uludag University, Bursa, Turkey; 2 Independent Researcher, Sabzevar, Iran; 3 Mathematics Education Department, Bursa Uludag University, Bursa, Turkey; 4 Centre for Artificial Intelligence Research and Optimisation, Torrens University Australia, Brisbane, Australia; 5 University Research and Innovation Center, Obuda University, Budapest, Hungary; 6 Faculty of Electrical Engineering and Computer Science, VSB – Technical University of Ostrava, Ostrava, Czech Republic

**Keywords:** Roller compacted concrete, Fly ash, Compressive strength, Splitting tensile strength, Artificial intelligence, Optimization technique, Engineering, Mathematics and computing

## Abstract

Roller compacted concrete (RCC) has gained prominence in the construction industry due to its durability, cost-effectiveness, and environmental benefits, particularly with the incorporation of high-volume fly ash (HVFA). However, traditional experimental approaches to evaluating RCC’s mechanical properties, such as compressive strength (CS) and splitting tensile strength (STS), are resource-intensive and time-consuming. To address these challenges, this study explores the application of artificial intelligence (AI), specifically artificial neural networks (ANN) and a hybrid ANN-Biogeography-Based Optimization (ANN-BBO) model, to predict the CS and STS of RCC. A dataset comprising 168 RCC mixtures, incorporating various material and process parameters, was analyzed. The ANN-BBO model demonstrated superior predictive accuracy compared to a standalone ANN, with R^2^ values exceeding 0.98 for both CS and STS, significantly reducing error margins. The findings highlight the effectiveness of AI-driven modeling in optimizing RCC mix designs, minimizing experimental costs, and enhancing the sustainability of concrete production. This research underscores the potential of integrating AI with optimization techniques to refine RCC performance assessment, which enables and facilitates more efficient and sustainable infrastructure development.

## Introduction

Roller compacted concrete (RCC) has emerged as a significant type of concrete in the construction industry due to its high durability and cost-effectiveness compared to conventional concrete^1,2^. RCC presents a multitude of benefits that significantly enhance both its production methodologies and the various applications to which it can be subjected. Among these numerous advantages are the notable elimination of the necessity for steel reinforcement, which not only simplifies the construction process but also leads to reduced material expenditures when compared to conventional concrete options, as well as an exceptional resistance to the extreme fluctuations in temperature associated with both scorching hot and frigid cold weather conditions, alongside an impressive capacity for rapid production. Furthermore, the overall production process associated with RCC is remarkably swifter and more efficient than that of conventional concrete, thereby offering a considerable competitive edge, particularly in the context of large-scale construction projects that demand timely and effective execution^3,4^. In summary, the unique characteristics and properties of RCC render it an increasingly appealing choice for engineers and contractors seeking innovative solutions in the field of modern construction practices.

The mechanical properties of RCC, particularly compressive strength and splitting tensile strength, are critical indicators of its performance in structural applications. These properties influence the design and safety of structures while also playing a crucial role in overall service life and maintenance costs^5^. In addition to the strength performance of concrete, the carbon footprint is recognized as a key parameter^6,7^. Particularly following the signing of the Paris Climate Agreement in 2015, issues such as the use of alternative materials to replace those that generate significant greenhouse gas emissions during production, as well as energy efficiency, have gained prominence^8^.

Environmental sustainability has emerged as a major global priority in today’s world. In this context, the construction industry plays a crucial role in developing innovative solutions aimed at reducing waste generation and enhancing the performance of materials used in infrastructure development. These advancements seek to minimize the carbon footprint of construction activities while also promoting the adoption of environmentally friendly practices that contribute to a more sustainable future.

In this regard, the use of pozzolans as a partial replacement for cement has been shown to reduce the carbon footprint of concrete. Fly ash, a byproduct of coal combustion, is recognized as a valuable supplementary material that enhances both the durability and strength of concrete while simultaneously mitigating its environmental impact. Incorporating fly ash into concrete mixtures not only improves the structural performance of concrete but also encourages the recycling of industrial waste. This approach aligns with circular economy principles and supports a more efficient resource management strategy^9–12^. However, in concretes with high fly ash content, potential effects on workability and setting times must be carefully considered. This necessitates appropriate adjustments in mix design to achieve optimal performance without compromising quality^13,14^.

A laboratory-based approach can provide definitive results when evaluating the compressive strength (CS) or splitting tensile strength (STS) of high-volume fly ash roller compacted concrete (HVFA-RCC)^15,16^. However, this approach may present various challenges. These challenges include time and natural resource consumption, the need for retesting due to varying material properties, the limited number of prepared and tested mixtures, and the necessity for extensive data analysis to derive meaningful conclusions^17,18^. For these reasons, manually conducting trials optimize RCC mixtures for specific properties (e.g., strength, durability) is labor-intensive and lacks the responsiveness of data-driven methods like AI-based models.

To overcome these challenges, innovative approaches that enhance testing processes, improve material efficiency, and promote sustainability in the construction industry are required. Advanced modeling and simulations enable predictive analyses that refine mix design, reduce the need for physical testing, and optimize performance. Machine learning and deep learning techniques are rapidly emerging as powerful tools capable of analyzing complex datasets, identifying patterns, and predicting the behavior of various material combinations under diverse conditions, thereby optimizing the development process. These technologies not only improve prediction accuracy but also support a more sustainable approach by minimizing waste and resource consumption throughout the construction lifecycle. In these methodologies, various algorithms are employed to evaluate the impact of different variables on construction outcomes, ultimately enhancing decision-making and increasing efficiency in project management. By leveraging the potential of machine learning, construction professionals can significantly reduce project timelines and costs while ensuring superior quality standards in materials and designs^19^.

Machine learning and deep learning, particularly artificial neural networks (ANN), play a crucial role in predicting concrete’s mechanical properties with high accuracy. Studies show that ANN can achieve correlation coefficients over 0.95 when applied to datasets with input parameters related to concrete composition and curing conditions^18^. Artificial intelligence (AI) techniques not only speed up the design process but also enable engineers to explore a wider range of material combinations, overcoming the limits of traditional methods. This shift supports sustainability in construction by optimizing resource use and minimizing waste^20^. As AI tools advance, they improve efficiency, reduce material waste, and lead to more customized concrete formulations for specific project needs^21^.

**Fig. 1:**
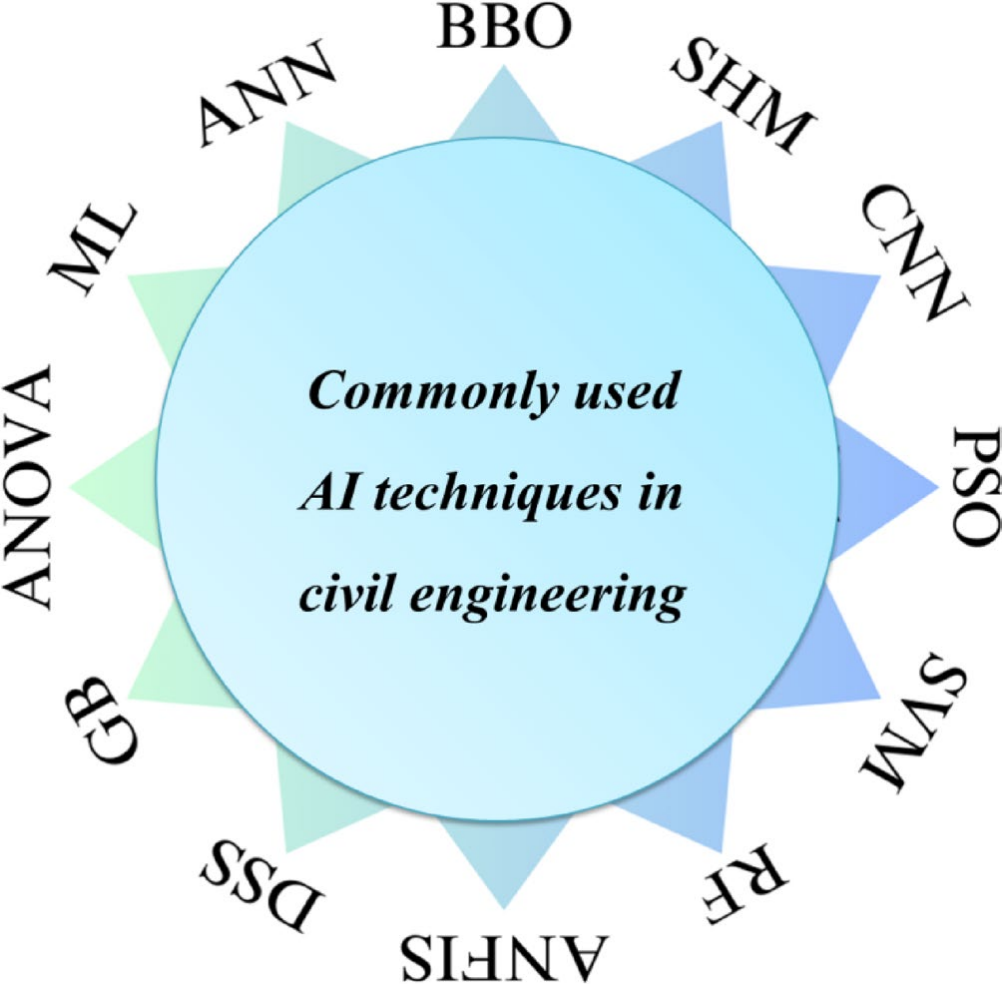
Commonly used AI models in civil engineering studies.

A review of the literature reveals a growing volume of research on modelling RCC. These studies emphasize the importance of understanding the mechanical properties of RCC through advanced modeling techniques. Table 1 lists AI modeling studies conducted on roller-compacted concrete and Fig. 1 presents commonly used AI techniues in civil engineering researches.

**Table 1:** AI modeling studies on RCC.

References	Products and wastes used in RCC mixtures	AI technique	Dataset	The best model for CS (R value)	The best model for STS (R value)
^22^	RAP & crumb rubber	*ANN *GA-ANN	56	GA&ANN (0.945)	–
^23^	Rice husk ash	*ANFIS	9	ANFIS (0.9986)	–
^24^	Macro-synthetic fibre	*ANOVA	15	ANOVA (0.9647)	–
^14^	High-volume fly ash, crumb rubber & nano silica	*ANOVA	16	ANOVA (0.9803)	ANOVA (0.9327)
^25^	-	*GEP (1,2–3)	235	GEP 1 (0.901)	–
^21^	-	*RF *M5r *M5p *CHAID	929	RF (0.986)	RF (0.991)
^17^	EAF slag & fly ash	*MRA *ANN *FL	53	FL (0.9817)	–
^26^	-	*ANN *PSO-ANN	500	PSO-ANN (0.922)	–
^27^	Crumb rubber & nano silica	*ANOVA	68	ANOVA (0.9849)	–
^28^	-	*ANN *RF *MARS *MARS-GOA *ELM *M5p	947	MARS-GOA (0.9327)	MARS-GOA (0.9322)
^29^	RAP	*LM-ANN *BR-ANN *SCG-ANN *2HL-ANN	83	ANN-BR (0.985)	–
^30^	–	*PSO-LightGBM *SVR *LM-ANN	408	PSO-LightGBM (0.9808)	–
^31^	Color pigment	*ML *GB *RF *SVM *ANN *BGG	239	ANN (0.9808)	–

To date, numerous AI-based prediction methods have been developed, and these methods continue to evolve in terms of accuracy, efficiency, and adaptability to various applications. Among these methods, artificial neural network (ANN) stand out as a powerful tool for modeling and predicting complex relationships within data. ANN have been widely used by researchers, particularly in assessing the mechanical properties of concrete. The ability of ANN to learn from data and improve prediction accuracy over time makes them a valuable method in civil engineering applications^32–34^. In this context, ANNs are employed to predict the properties of eco-friendly concretes. Moreover, they enable engineers to minimize the environmental impact of concrete while allowing for the development of optimal formulations that maintain structural integrity and performance parameters^35–37^.

The limitations of existing AI techniques are often linked to their dependence on large datasets for training, which can be difficult to obtain in specialized areas of civil engineering, potentially leading to inaccuracies and biases in predictions. Furthermore, the scarcity of data in rare engineering scenarios, along with concerns over data privacy and security, adds complexity to their application. Many AI tools, particularly deep learning neural networks, operate as “black boxes” with limited interpretability, which presents challenges in engineering analysis and design, where transparency in decision-making is critical. Ongoing research is focused on improving model interpretability to provide clearer insights into their reasoning. The effective application of AI in engineering necessitates expertise in both AI and civil engineering, as the development and validation of AI tools require specialized knowledge. Engineers must also be adequately trained to apply these tools appropriately and interpret the results with precision^38,39^.

To overcome these challenges, researchers are exploring innovative approaches to enhance data collection and model training processes, such as the use of synthetic data generation and transfer learning techniques. Additionally, binary AI models are being employed to streamline decision-making by simplifying complex data interpretations, allowing engineers to focus on the critical elements of their projects without being overwhelmed by intricate algorithms. These advancements aim to strengthen the robustness of AI models, enabling them to perform more reliably in scenarios where real-world data is limited or challenging to obtain. As a result, the integration of these techniques not only improves model performance but also expands the applicability of AI solutions across a wide range of civil engineering projects^38,40^.

In RCC modeling studies, most of the proposed models are independent of each other, and hybrid models integrated with meta-heuristic optimization techniques have not yet been fully evaluated. Moreover, a review of RCC modeling studies reveals that the number of studies focusing on splitting tensile strength, one of the most critical parameters of RCC, is relatively limited^27,41^. These gaps present a significant opportunity for new research that, through the integration of advanced optimization methods, can enhance the predictive accuracy and reliability of ANN models in RCC applications. Additionally, the integration of ANN with Biogeography-Based Optimization (BBO) emerges as an innovative approach to improving prediction accuracy by optimizing the parameters of the neural network model^42,43^. Thus, the lack of ANN-BBO artificial intelligence model, which has not been applied in RCC studies before, will be overcome and the analysis of the splitting tensile strength will contribute to the limited number of splitting tensile strength modeling in the literature.

This study aims to develop models for predicting the compressive strength and splitting tensile strength of RCC using the frameworks of ANN model and ANN-BBO, a model that has not been used in RCC studies before. Data obtained from a study on the formation of interlayer cold joint in RCC having high volume fly ash, aiming aims to assess mechanical, permeability, and freeze–thaw properties to prevent cold joint^44^, and were modeled in this research. Additionally, this study seeks to evaluate the performance of the developed models and examine their implications for future engineering applications. The findings are expected to contribute to the existing body of knowledge and provide valuable insights for future research on optimizing concrete properties. Ultimately, the results are anticipated to highlight the effectiveness of combining AI with optimization techniques, leading to more efficient and sustainable applications in civil engineering.

## Data definition

In this study, a dataset comprising 168 different RCC mixtures was analyzed to predict the compressive strength (CS) and splitting tensile strength (STS) of RCC^44^. The dataset was collected from an extensive experimental study focused on preventing cold joint formation in high-volume fly ash roller compacted concrete (HVFA-RCC). The data acquisition process involved laboratory testing of RCC specimens with varying material compositions and curing conditions. The data collection process was conducted in multiple stages to ensure accuracy and reliability. The following steps outline the methodology used:

### Material selection and preparation

The materials incorporated in RCC mixtures comprised cement, fly ash, fine aggregates (0–5 mm), coarse aggregates (5–15 mm and 15–25 mm), water, set retardant admixture, adherence-enhancing additives, and interlayer mortar application, with their selection being driven by factors such as their widespread availability and prevalent utilization in typical RCC applications.

### Mix design and proportioning

A total of 168 distinct RCC mix designs were formulated, each incorporating varying proportions of cementitious materials, aggregates, and additives. These mix designs were developed to assess the influence of material variations on compressive strength (CS) and split tensile strength (STS), with the goal of optimizing sustainability through elevated levels of fly ash replacement.

### Specimen casting and curing

The concrete mixtures were prepared in accordance with standardized procedures to maintain consistency. To replicate field conditions, the fresh RCC mixtures were compacted using a vibrating hammer. Subsequently, the specimens were cured under controlled temperature and humidity conditions, simulating real-world environmental exposure.

### Strength testing

After the curing period, the mechanical properties of the RCC were evaluated. Compressive strength (CS) was measured using cubic specimens subjected to compression following EN 12390-3 standards, while splitting tensile strength (STS) was evaluated using EN 12390-6 procedures. These tests yielded essential data for modeling the strength characteristics of RCC.

### Data recording and preprocessing

The results from mechanical testing were meticulously documented, with outliers being identified and excluded to ensure the reliability of the data. Key parameters, including cement (*X*_1_), fly ash (*X*_2_), aggregate proportions (*X*_3_–*X*_5_), water content (*X*_6_), maximum dry unit weight (*X*_7_), Vebe time (*X*_8_), admixture dosages (*X*_9_–*X*_10_), interlayer mortar application (*X*_11_), and waiting time between layers (*X*_12_), were considered as input variables. Compressive strength (CS) and splitting tensile strength (STS) values were designated as the dependent output variables.

### Normalization and statistical analysis

**Fig. 2:**
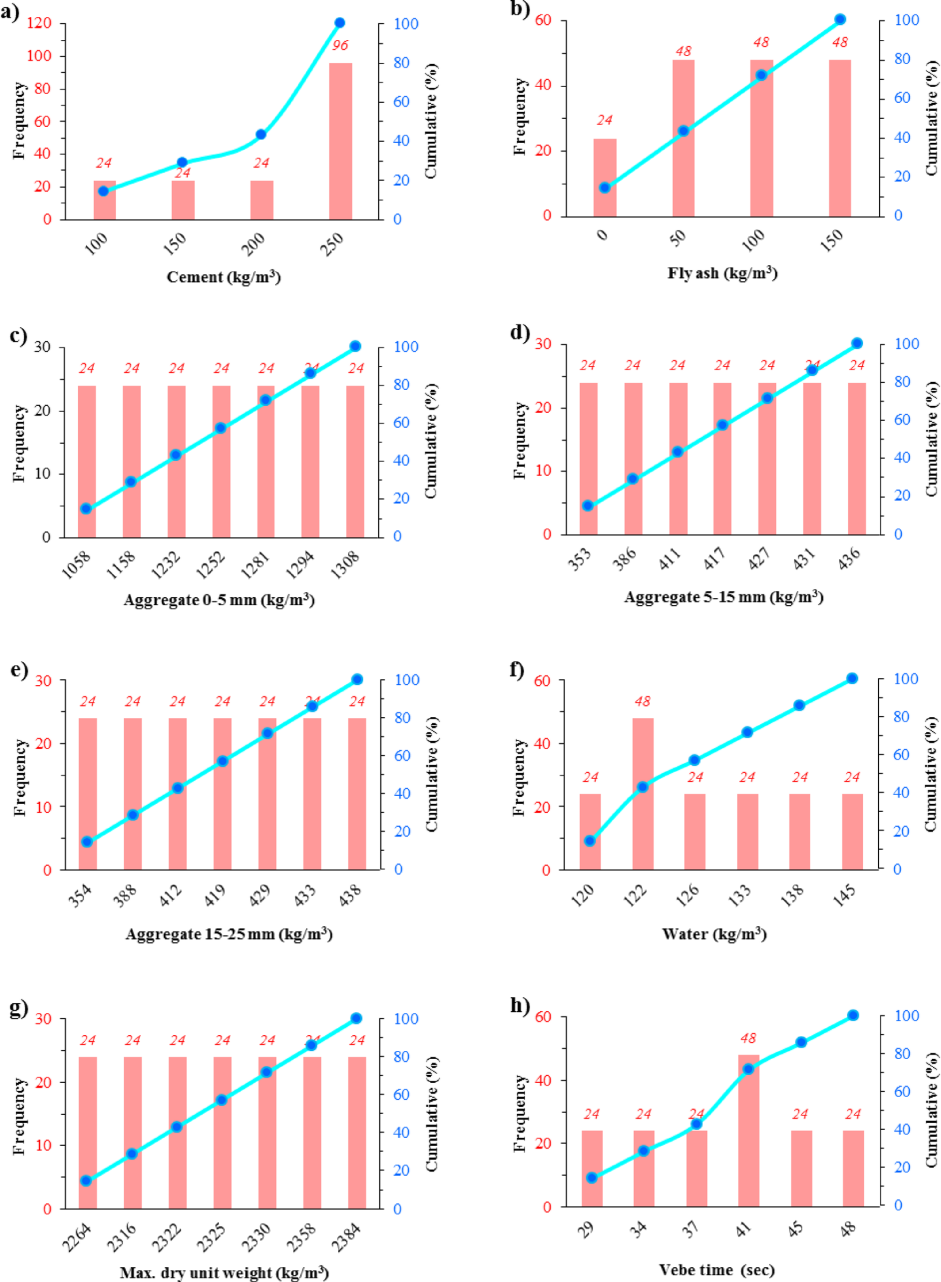

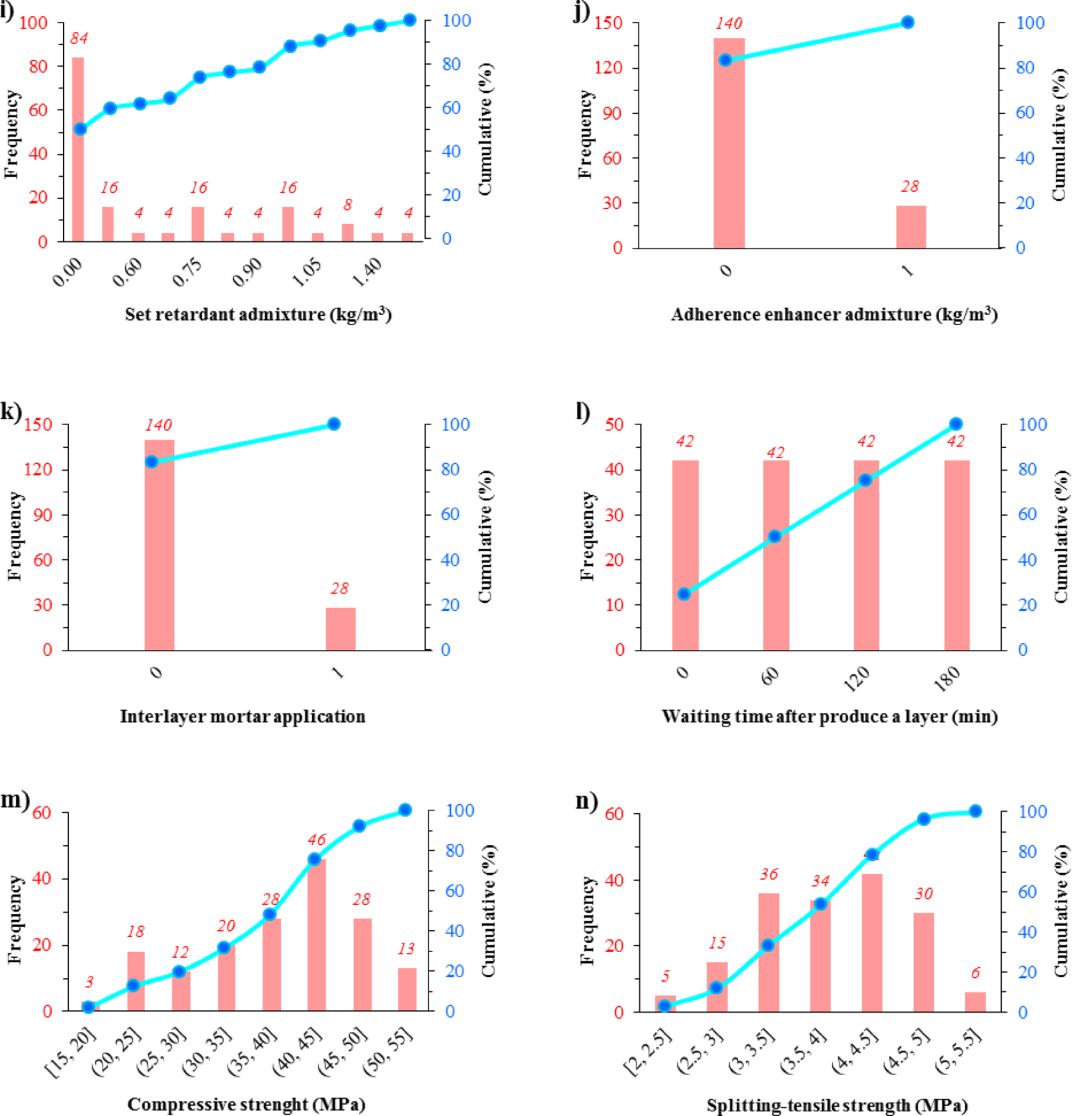
Histograms of the frequency of input (a-l) and output (m & n) parameters.

To enable effective AI modeling, all input parameters were normalized using the Z-score method, ensuring that discrepancies in scale or units did not introduce bias into the model’s performance. Descriptive statistics, including range, mean, standard deviation, skewness, and kurtosis, were calculated to evaluate the data distribution, as detailed in Fig. 2 and Table 2.

**Table 2:** Descriptive statistics of the variables.

ID No.: Variables	Unit	Min–Max	Mean	Standard deviation	Kurtosis	Skewness
*X*_1_: Cement	kg/m^3^	100–250	207.14	56.24	− 0.76	− 0.89
*X*_2_: Fly ash	kg/m^3^	0–150	85.71	51.51	− 1.14	− 0.19
*X*_3_: Aggregate (0-5 mm)	kg/m^3^	1058–1308	1226.14	82.75	− 0.24	− 1.02
*X*_4_: Aggregate (5-15 mm)	kg/m^3^	353–436	408.71	27.44	− 0.24	− 1.02
*X*_5_: Aggregate (15-25 mm)	kg/m^3^	354–438	410.43	27.72	− 0.22	− 1.03
*X*_6_: Water	kg/m^3^	120–145	129.43	8.78	− 1.11	0.59
*X*_7_: Max. dry unit weight	kg/m^3^	2264–2384	2328.43	34.48	− 0.19	− 0.25
*X*_8_: Vebe time	sec	29–48	39.29	6.02	− 0.94	− 0.26
*X*_9_: Set retardant admixture	kg/m^3^	0–1.6	0.44	0.49	− 0.97	0.58
*X*_10_: Adherence enhancer admixture	kg/m^3^	0–1	0.17	0.37	1.27	1.81
*X*_11_: Interlayer mortar application	–	0–1	0.17	0.37	1.27	1.81
*X*_12_: Waiting time after produce a layer	min	0–180	90.00	67.08	− 1.36	0.00
*Y*_*1*_: Compressive strength	MPa	17.8–54.0	38.52	8.84	− 0.61	− 0.51
*Y*_*2*_: Splitting-tensile strength	MPa	2.2–5.2	3.91	0.72	− 0.83	− 0.26

The comprehensive and systematic approach to data collection ensured the robustness and precision of the dataset, which establishes a reliable foundation for predictive modeling through artificial intelligence techniques. Moreover, the integration of machine learning methodologies in analyzing RCC properties facilitates a more efficient exploration of the relationships between material characteristics and mechanical performance.

**Fig. 3:**
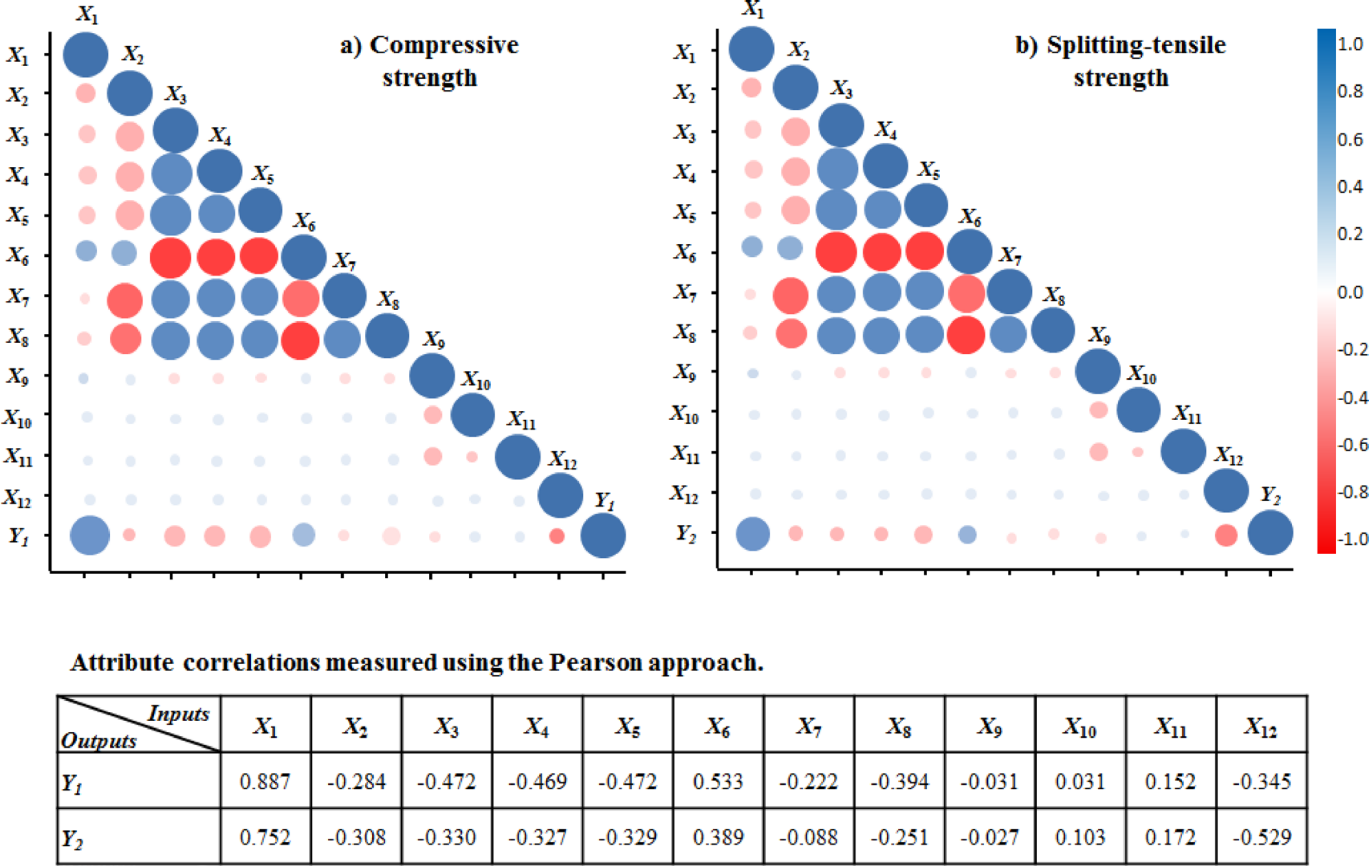
Correlation matrix of parameters for (**a**) CS and (**b**) STS.

Figure 3 shows the correlation matrix for 12 inputs to highlight the distribution of pairwise correlation coefficients. In this regard, a linear correlation between inputs (*X*_1_ − *X*_12_) and outputs (CS (*Y*_1_) and STS (*Y*_2_)) has been performed. The coefficient values range from − 1 to 1, with a value of 1 indicating a strong positive relationship and a value of − 1 indicating a strong negative relationship. The highest correlation coefficient between the parameters and both CS and STS outputs is for the cement parameter (*X*_1_), which is less than 0.9, and the reason for this is the undeniable role of cement as the main parameter in concrete materials. For both outputs, the absolute values of the correlation coefficients are not close to 1, indicating that multicollinearity is not a concern in this analysis.

## Overview of AI techniques used

### Artificial neural network (ANN)

**Fig. 4:**
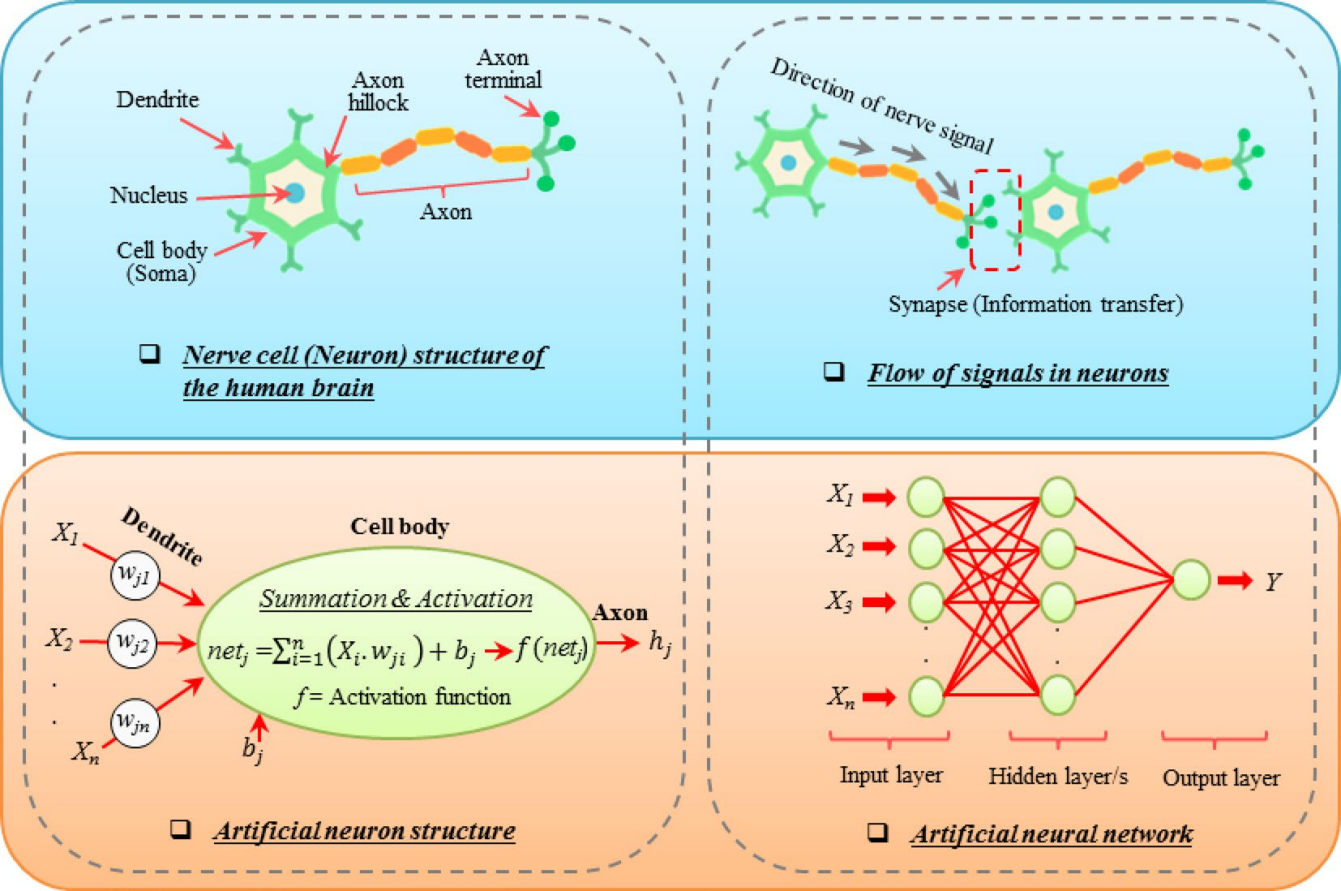
The function of neurons in the human brain and its inspired ANN structure.

ANNs are computational models inspired by the way signals flow through the structure of a nerve cell (neuron) in the human brain. Indeed, they simulate the interaction between incoming signals and resulting output. Typically, an ANN structure includes three main components: (i) an input layer that receives input signals and data from external sources, (ii) one or more hidden layers that are located between the input and output layers and process the information internally, and (iii) an output layer that provides the results of the model processing. The number of neurons in these layers can differ and is influenced by various factors, with the neurons in each layer remaining unconnected to one another^45^. The input and output variables determine the number of input and output neurons required for the model. The number of hidden layer neurons can vary, and it is very important to consider their optimal number to achieve accurate results^46,47^. In summary, a schematic representation of the model structure and its formulation can be found in Fig. 4 and Eqs. (1) and (2), respectively.

1 $${net}_{j}=\sum_{i=1}^{n}\left({X}_{i}{.w}_{ji}\right)+{b}_{j}$$

2 $${h}_{j}=f{(net}_{j})$$

where net_*i*_: Net input signal, *X*_*i*_ (*i* = 1,2,…,*n*): Set of input parameters, w_*ij*_: Connection weights, b_*j*_: Bias term, h_*j*_: Neuron’s output, *f*: Activation function.

The adaptability of the ANN enables it to adjust its structure during the learning process based on the data it encounters. By doing so, it identifies and models intricate connections between input and output variables, enabling it to solve complex problems^48^.

### Biogeography-based optimization (BBO)

**Fig. 5:**
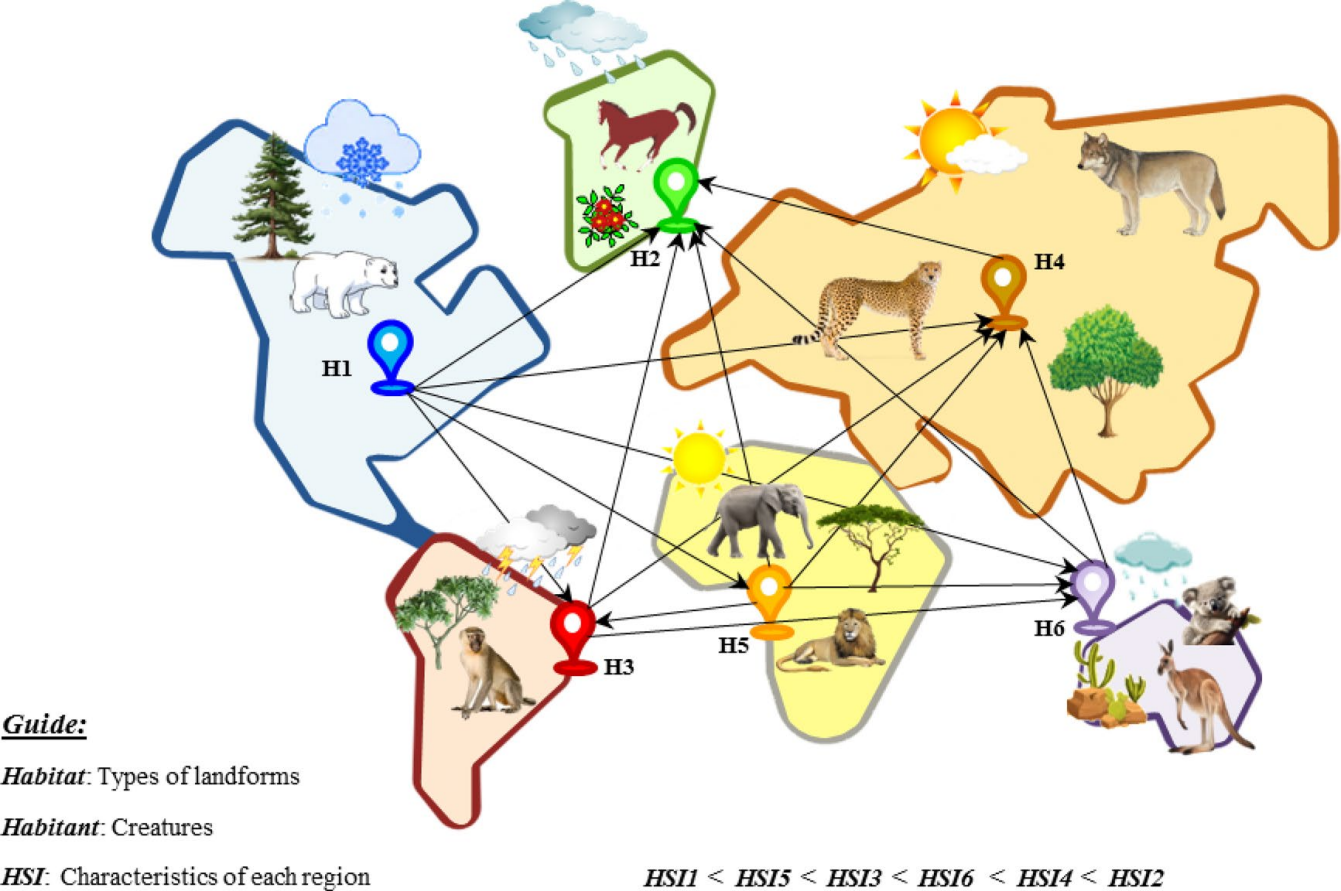
The BBO method, inspired by the migration and mutation processes observed in biogeographic species.

BBO draws inspiration from the concept of biogeography, which describes how species are distributed across ecosystems, as well as their relocation and extinction^49^. In biogeography, islands are small ecosystems consisting of populations of diverse species that are isolated from other habitats. Islands capable of supporting a diverse array of species are classified as having a habitat suitability index (HSI). The HSI of an island is influenced by factors such as climate, soil fertility, and vegetation. These environmental factors are referred to as suitability index variables (SIVs) that contribute to determining the island’s HSI. Habitat areas with high HSI (representing favorable conditions, i.e., a good solution) tend to host larger populations, whereas habitats with low HSI (indicating poor conditions, i.e., an inadequate solution) can experience higher migration rates and reduced inhabitants. Thus, good solutions are more inclined to share SIV with those having a low HSI and vice versa. The two main operators in the optimization process are migration and mutation (Eqs. 3–5). The migration operation enables the discovery of new areas of the search space by exchanging solutions between habitats using emigration and immigration rates. The mutation operation plays a crucial role because an abrupt change in the species impacts the solution coefficients. This avoids the problem of getting trapped in a local optimum^50^. Figure 5 illustrates a schematic representation of the biogeographically inspired BBO model.

3 $${\text{Emigration }}\;{\text{rate}}_{h} = {\text{Emigration}}_{max} \frac{h}{{h_{max} }}$$

4 $${\text{Immigration }}\;{\text{rate}}_{h} = {\text{ Immigration}}_{max} \left( {1 - \frac{h}{{h_{max} }}} \right)$$

5 $${\text{Mutation }}\;{\text{rate}}_{h} = {\text{Mutation}}_{max} \left( {1 - \frac{{P_{h} }}{{P_{max} }}} \right)$$

where *h*: No. of current habitants, *h*_*max*_: Maximum of No. of habitants that the habitat can support, *P*_*h*_: Mutation probability of the *h*th habitat, *P*_*max*_: argmax(*P*_*h*_).

## Research methodology

**Fig. 6:**
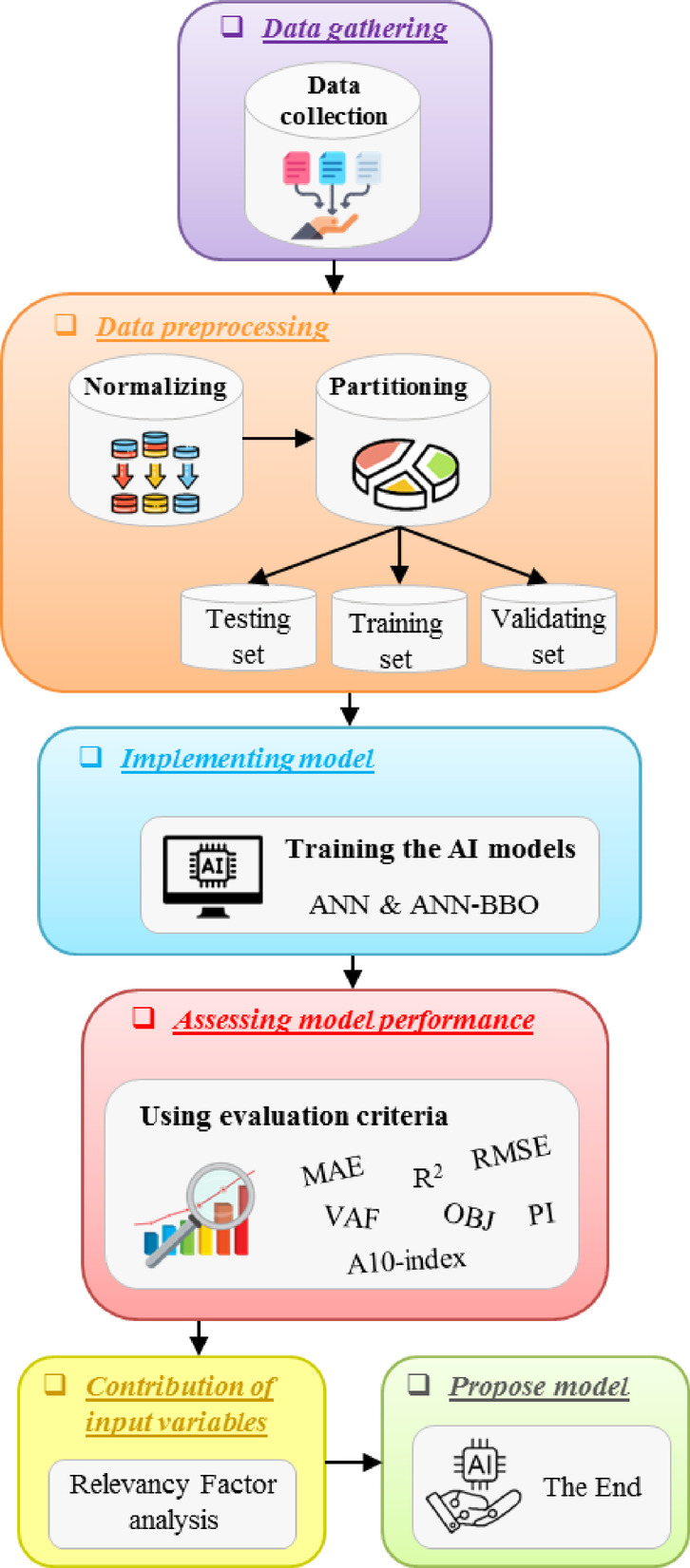
Flowchart of the research process.

This section seeks to provide a thorough description of the research process undertaken to propose an AI framework for modeling the CS and STS properties of RCC. To achieve this, two AI model frameworks, single ANN and ANN integrated with BBO, are proposed. Comparing these models offers valuable insights into the effectiveness of the hybrid approach versus the single model. The BBO metaheuristic optimization technique is applied to identify optimal variables, ensuring that the prediction model enhances accuracy while minimizing errors. At a glance, Fig. 6 illustrates the workflow in this study.

### Data and model preparation

Data preprocessing is considered one of the initial and essential steps in modeling because it significantly increases data quality by removing data biases caused by scale or unit differences among different parameters, thereby improving the effectiveness of model development. In this regard, we normalized all parameter data with the Z-score approach according to Eq. (6).

6 $$Z=\frac{X- \mu }{\sigma }$$

where *X*: Measured value of each parameter, *μ*: Mean value of the data for each parameter, *σ*: Standard deviation of the data for each parameter.

When assessing AI models, a key question is whether the proposed model is the best one within its hypothesis space, particularly in terms of generalization to new, unseen data. The answer to this question is closely tied to data partitioning, which is a critical step in the modeling process. In order for our model to cover this generalizability, we use the three-way hold-out method to partition the data. For this purpose, in both the CS and STS categories, all data (168 data records) are randomly split into three sets: train, validate, and test, with proportions of 70% (118), 15% (25), and 15% (25), respectively. Each of these sets plays a specific role: Training the model and learning the key patterns in the data by training set, preventing the overfitting by validation set, and evaluating the generalizability performance by testing set. Given the superior learning performance of the Levenberg–Marquardt algorithm reported in the studies^51,52^, this algorithm is considered the learning algorithm. Furthermore, the hyperbolic tangent function, as described in the most cited book by Haykin^53^, is utilized as the transfer function. To determine the number of hidden layers, a highly cited study^54^ pointed out that it is not always necessary to have two or more layers for real-valued functions. For continuous functions, one hidden layer is sufficient. This is ensured by the universal approximation theorem^55^. Any continuous function can be approximated to arbitrary accuracy by a network with a single hidden layer, for sufficiently many neurons in the hidden layer. Further, according to the type of activation function used in this study (i.e. hyperbolic tangent), the performance of networks with many hidden layers can be sensitive to the initialisation of the weights. In addition, vanishing- or exploding-gradient problem aggravated the problem when the network has multiple hidden layers^54^. Based on these considerations, this study employs an architecture with only one hidden layer. With 12 input parameters (*X*_1_–*X*_12_) and one output parameter (either *Y*_1_ or *Y*_2_), the model follows a 12-*Hidden*_neuron_ − 1 arrangement. A thorough analysis is necessary to define the final model architecture (i.e., *Hidden*_neuron_), which will be discussed in the initial part of the results section.

### Model performance evaluation

This subsection aims to present the statistical measures employed to examine the performance of the developed models. A detailed list of these measures is provided in Table 3. Calculating and comparing these measures helps to identify an effective model.

**Table 3:** The statistical measures.

Criteria (Abbreviation)	Equation	Range (Ideal)
Coefficient of determination (R^2^)	$\left( {\frac{{\mathop \sum \nolimits_{{{\text{i}} = 1}}^{N} \left( {Y_{mi} - \overline{{Y_{m} }} } \right)\left( {Y_{pi} - \overline{{Y_{p} }} } \right)}}{{\sqrt {\left[ {\mathop \sum \nolimits_{{{\text{i}} = 1}}^{N} \left( {Y_{mi} - \overline{{Y_{m} }} } \right)^{2} } \right]\left[ {\mathop \sum \nolimits_{{{\text{i}} = 1}}^{N} \left( {Y_{pi} - \overline{{Y_{p} }} } \right)^{2} } \right]} }}} \right)^{2}$	(0, 1) 1
Mean absolute error (MAE)	$\frac{1}{N}\mathop \sum \limits_{{{\text{i}} = 1}}^{N} \left| {Y_{mi} - Y_{pi} } \right|$	(0, $+ \infty$) 0
Root mean squared error (RMSE)	$\sqrt {\frac{1}{N}\mathop \sum \limits_{{{\text{i}} = 1}}^{N} \left( {Y_{mi} - Y_{pi} } \right)^{2} }$	(0, $+ \infty$) 0
Nash–Sutcliffe efficiency (NSE)	$1 - \frac{{\mathop \sum \nolimits_{{{\text{i}} = 1}}^{N} \left( {Y_{mi} - Y_{pi} } \right)^{2} }}{{\mathop \sum \nolimits_{{{\text{i}} = 1}}^{N} \left( {Y_{mi} - \overline{{Y_{m} }} } \right)^{2} }}$	($- \infty$, 1) 1
Performance index (PI)	$\frac{{{\text{RMSE}}}}{{\overline{{Y_{m} }} \left( {1 + {\text{R}}} \right)}}$	(0, 1) 0
Variance Accounted For (VAF)	$\left( {1 - \frac{{var\left( {Y_{m} - Y_{p} } \right)}}{{var\left( {Y_{m} } \right)}}} \right) \times 100$	(0, 100) 100%
Objective function (OBJ)	$$\begin{aligned} & \left( {\frac{{N_{Tr} }}{N}.\frac{{RMSE_{Tr} + MAE_{Tr} }}{{R_{Tr}^{2} + 1}}} \right) \\ & \quad + \left( {\frac{{N_{Va} }}{N}.\frac{{RMSE_{Va} + MAE_{Va} }}{{R_{Va}^{2} + 1}}} \right) \\ & \quad + \left( {\frac{{N_{Te} }}{N}.\frac{{RMSE_{Te} + MAE_{Te} }}{{R_{Te}^{2} + 1}}} \right) \\ \end{aligned}$$	(0, $+ \infty$) 0
A10-index	$\frac{{{\text{m}}10}}{N}$	(0, 1) 1

*N*, *N*_*Tr*_, *N*_*Va*_, and *N*_*Te*_ = The number of total, training, validating, and testing data, respectively.

${{Y}_{m}}_{i}$ and ${{Y}_{p}}_{i}$ = The measured and predicted strength of the *i*th data, respectively.

$\overline{{Y }_{m}}$ and $\overline{{Y }_{p}}$ = The averages of the measured and predicted strength of the *i*th data, respectively.

m10 = The number of data in which their ${Y}_{m}$/${Y}_{p}$ ratio fits in the range of 0.90–1.10.

## Results and discussion

### Finalizing the model’s architecture

The goal of this subsection is to achieve the final architecture of the model, i.e., the appropriate number of *Hidden*_neuron_. To accomplish this, the models are evaluated with different arrangements, ranging from 10 to 20 neurons. For consistency in comparison, all models are implemented using the same dataset. The statistical measures (R^2^ and RMSE) are calculated for each model and ranked based on the quality of the responses. Finally, the model with the highest total assigned ranks is considered the best model. Table 4 lists the results of this assessment. Based on these results, the model with 14 *Hidden*_neuron_ has the highest ranking score, representing the superior performance of the model compared to other neuron architectures. Accordingly, the 12-14-1 architecture is selected for the model.

**Table 4:** Performance and ranking of models with different No. of *Hidden*_neuron_.

No. of *Hidden*_neuron_	Value				Rank				Total ranking score
	CS		STS		CS		STS		
	R^2^	RMSE	R^2^	RMSE	R^2^	RMSE	R^2^	RMSE	
10	0.927	2.842	0.895	0.547	3	4	2	1	7
11	0.966	1.614	0.962	0.153	5	5	7	8	25
12	0.979	1.360	0.928	0.212	8	7	5	6	26
13	0.986	0.965	0.976	0.111	10	10	9	9	38
14	0.988	0.942	0.980	0.097	11	11	10	11	43
15	0.982	1.117	0.982	0.102	9	9	11	10	39
16	0.972	1.274	0.975	0.196	7	8	8	7	30
17	0.969	1.443	0.955	0.224	6	6	6	5	23
18	0.952	2.852	0.916	0.356	4	3	4	4	15
19	0.914	3.361	0.903	0.428	2	2	3	3	10
20	0.892	3.420	0.888	0.431	1	1	1	2	5

The ranking approach assigns the highest rank to the highest R^2^ value and the lowest RMSE value.

Table 5 summarizes the settings of selected parameters to present the information more effectively.

**Table 5:** Settings of selected parameters of ANN and BBO.

Model	Parameter		Setting
ANN	Dataset partitioning (%-No. of data)	Train	70%–118
		Validate	15%–25
		Test	15%–25
	No. of input parameter		12
	No. of hidden layer/nodes		1/14
	Learning algorithm		Levenberg–Marquardt
	Transfer function		Hyperbolic tangent
	Max. no. of epochs		500
BBO	Population size		100
	Max emigration rate		1
	Habitat modification		1
	Mutation probability		0.005

This analysis emphasizes the critical role of optimal parameter selection in maximizing the predictive performance of statistical models in civil engineering while simultaneously underscoring the necessity of rigorous validation procedures to guarantee their reliability in informing decision-making and influencing project outcomes in practical applications, as effective parameter tuning not only refines model precision but also enhances overall efficiency and sustainability in engineering projects, ultimately fostering superior resource management, minimizing material wastage, and driving cost-effective solutions.

### Regression plots

**Fig. 7:**
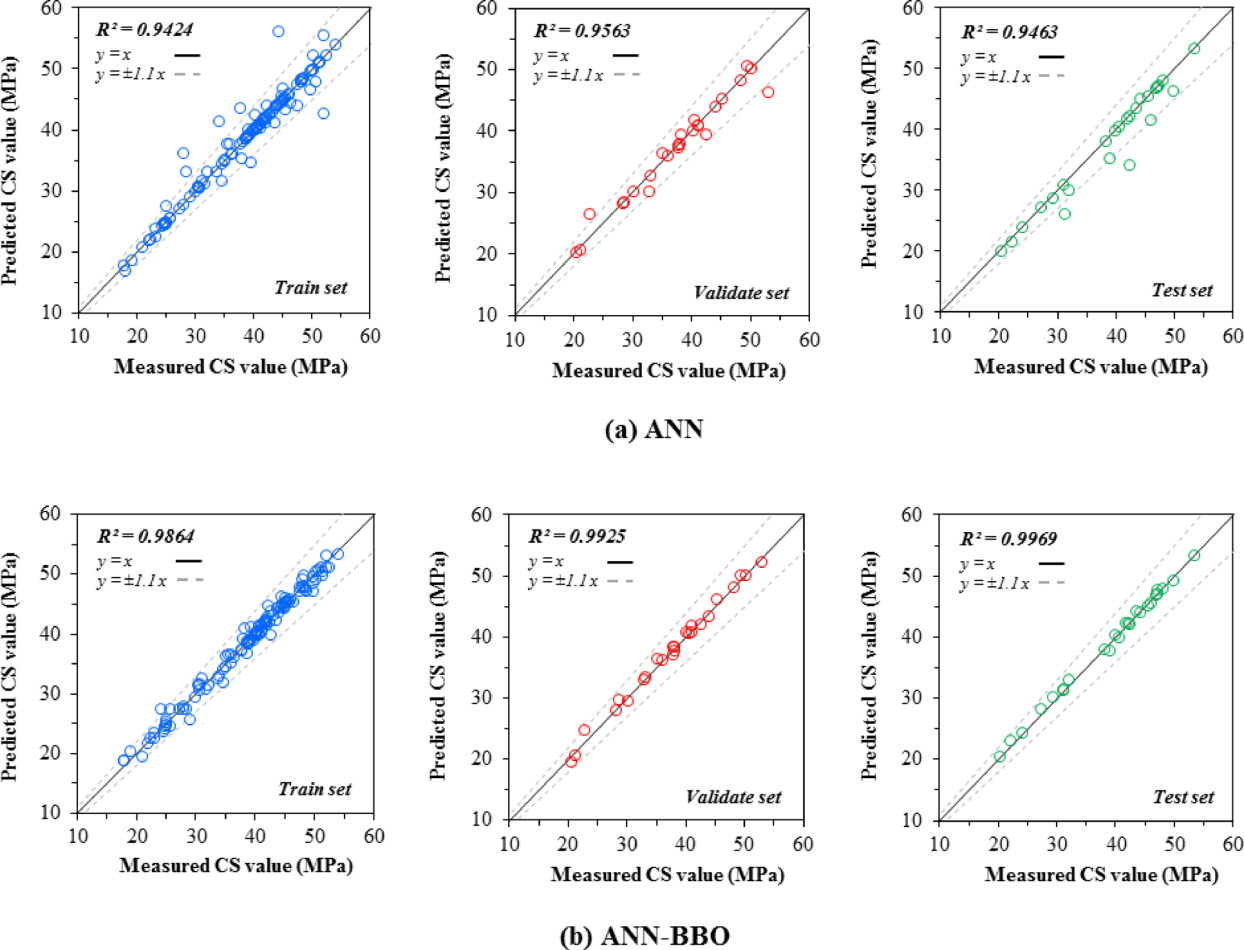
The measured CS vs. predicted CS values obtained by (a) ANN and (b) ANN-BBO models.

**Fig. 8:**
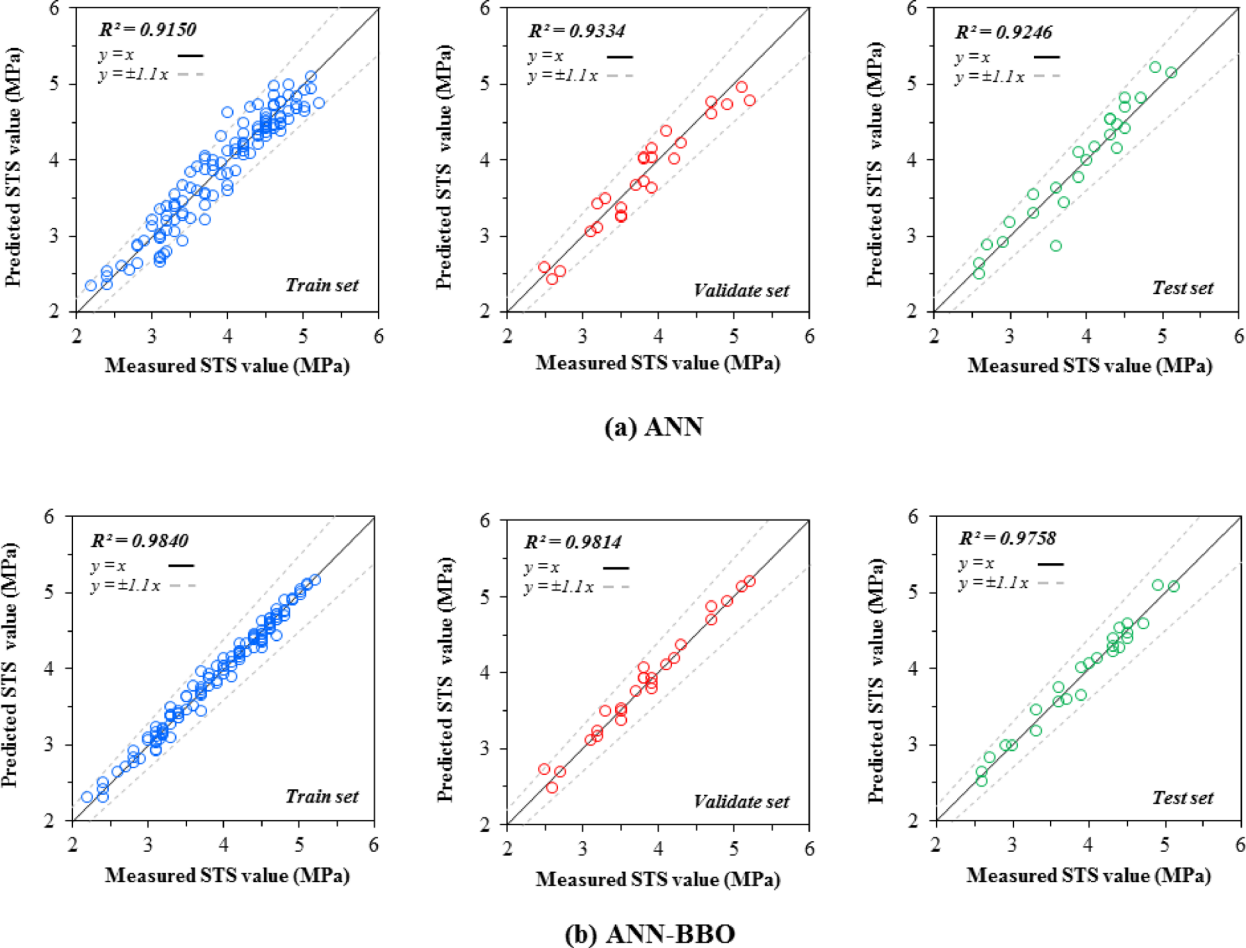
The measured STS vs. predicted STS values obtained by (**a**) ANN and (**b**) ANN-BBO models.

The correlation between the measured and predicted values of CS and STS in three datasets—train, validation, and test—is depicted in Figs. 7 and 8, respectively. As can be seen from Fig. 7, the ANN model can estimate the recorded CS data in the train, validate, and test datasets with R^2^ values of 0.9424, 0.9563, and 0.9463, while the corresponding values for the ANN-BBO model are 0.9864, 0.9925, and 0.9969, respectively. The ANN-BBO model exhibits a stronger correlation, meaning its data points align more closely with the best-fitting regression line (i.e., *y* = *x*) compared to those of the single ANN model. Additionally, in modeling the STS (Fig. 8), the hybrid model demonstrated its ability to accurately match most of the predicted responses with the actual values. This is evidenced by the concentration of data points in the results provided by the hybrid model (ANN-BBO) within the area between the black dotted lines in the plots, which indicate a ± 10% deviation from the *y* = *x* line. Accordingly, it can be concluded from the comparison of the measured vs. predicted plots that the ANN-BBO model demonstrates outstanding performance in estimating the CS and STS of RCC.

This performance underscores the transformative potential of hybrid models in enhancing predictive accuracy and reliability within civil engineering applications, fostering more efficient design methodologies and refined structural assessments. At the same time, these models streamline the design phase, reduce costs, and optimize resource allocation in engineering projects, highlighting the importance of integrating cutting-edge computational techniques with conventional engineering practices to drive safer and more sustainable infrastructure development. The growing adoption of hybrid models in civil engineering can lead to a paradigm shift, encouraging continued research and innovation aimed at optimizing materials and construction methodologies for future advancements.

### Error histogram plots

**Fig. 9:**
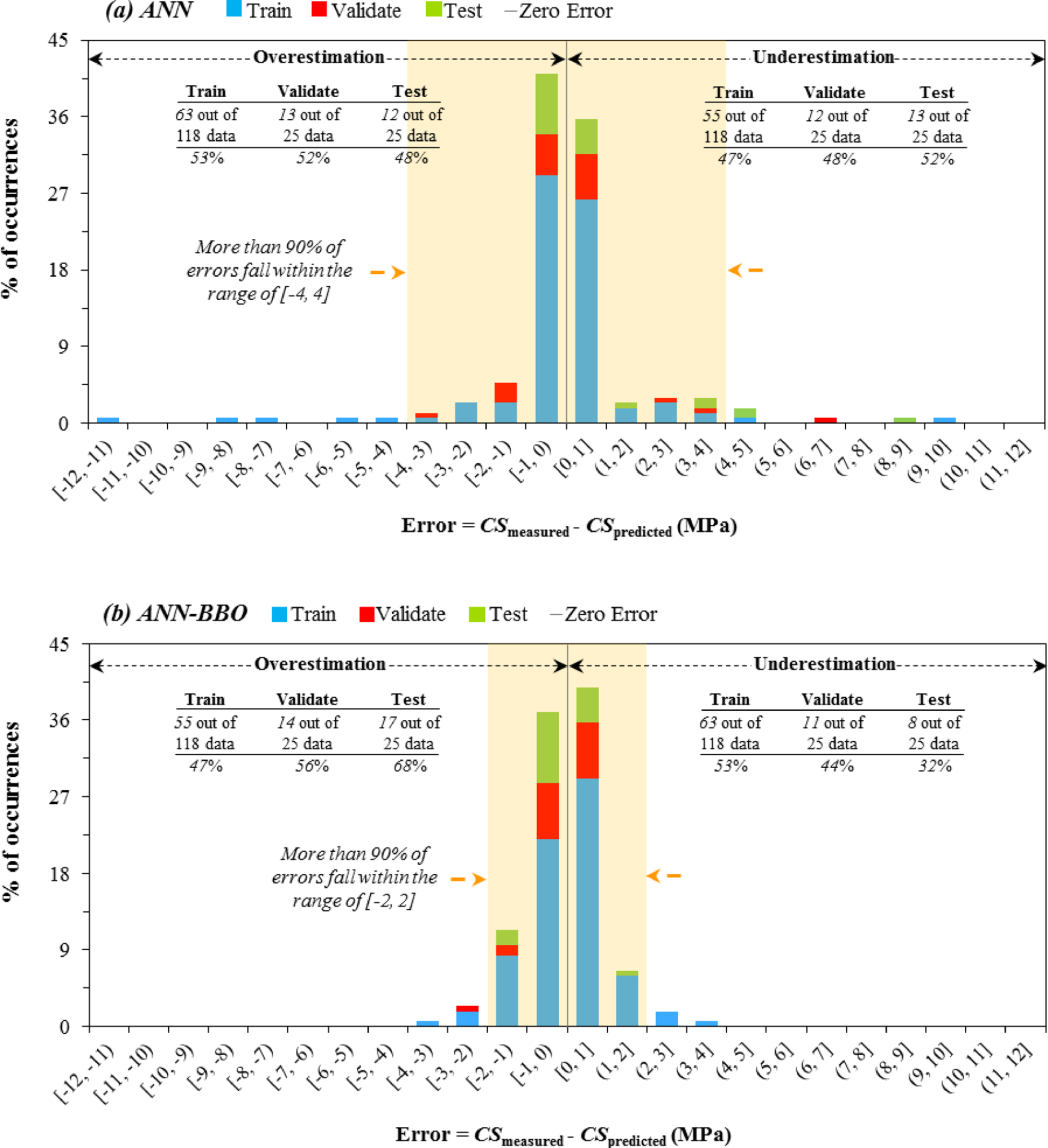
Error histogram for CS modeling with (**a**) ANN and (**b**) ANN-BBO models.

**Fig. 10:**
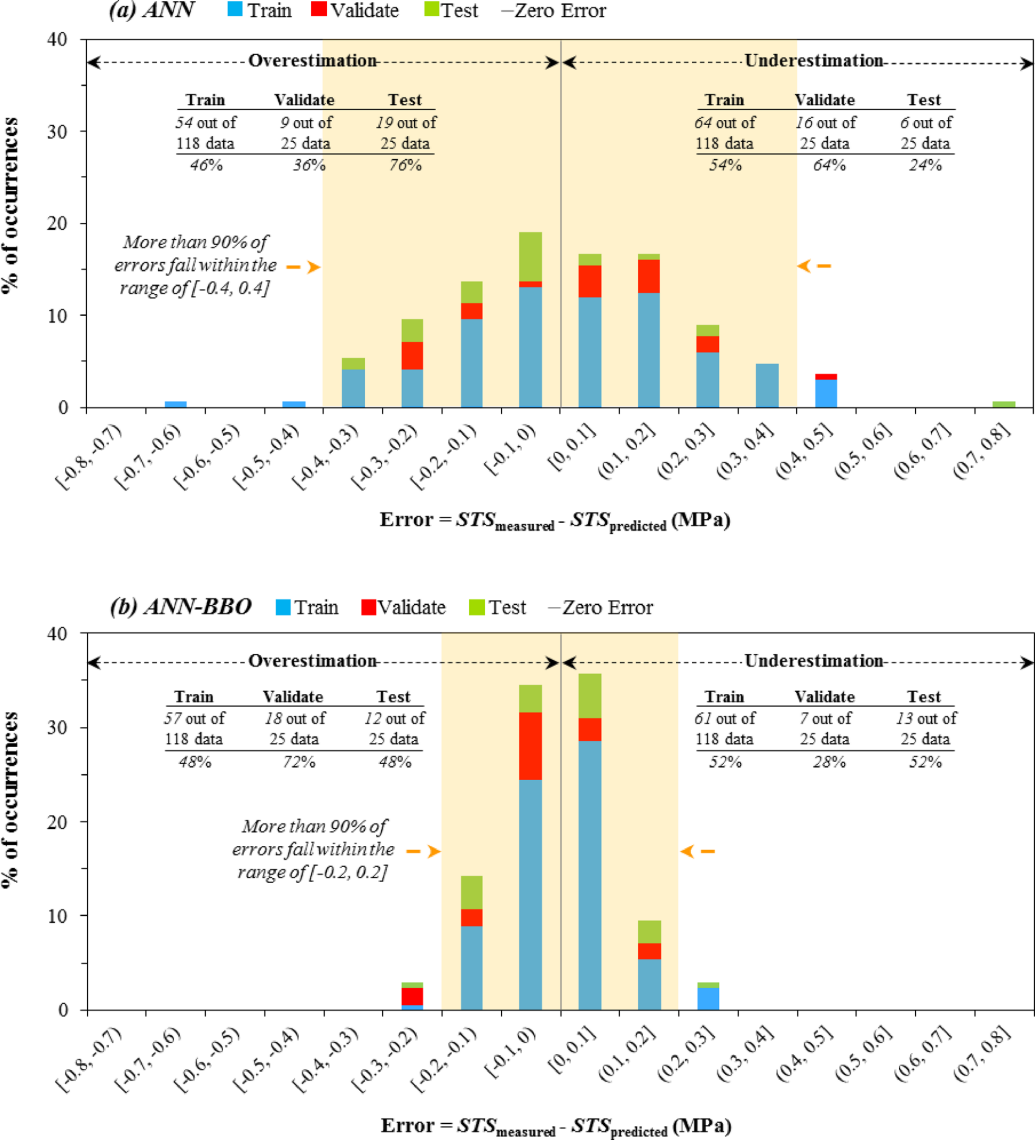
Error histogram for STS modeling with (**a**) ANN and (**b**) ANN-BBO models.

The histogram plots in Figs. 9 and 10 illustrate the performance error for each dataset, along with its occurrence percentage, in modeling CS and STS, respectively. This would allow for a more detailed examination of the distribution and range of errors in the performance of the proposed models. An evaluation of the CS prediction error histogram for the ANN-BBO model (Fig. 9b) reveals that over 90% of the prediction errors—specifically, 159 out of 168 data records (approximately 94%)—lie within the range of [− 2 MPa, 2 MPa], whereas the errors for the ANN model (Fig. 9a) span a broader range of [− 4 MPa, 4 MPa]. A similar trend is observed in the STS results (Fig. 10), where the ANN-BBO model estimated over 90% of the data within the interval [− 0.2 MPa, 0.2 MPa], demonstrating substantially greater accuracy than the ANN model with a broader range of [− 0.4 MPa, 0.4 MPa]. This indicates that the ANN-BBO model is capable of providing more accurate predictions with a narrower range of errors compared to the ANN model. The results inferred from the error histograms are consistent with those presented in the previous subsection, confirming the more reliable performance of the ANN-BBO model in predicting the strength properties of RCC compared to the single ANN model.

This enhanced predictive capability arises from the hybrid approach that combines ANNs with a BBO algorithm, which effectively fine-tunes model parameters, thereby improving overall accuracy. In the context of civil engineering applications, this advancement holds considerable potential for optimizing design processes and ensuring the structural integrity of reinforced concrete components under diverse load conditions. By leveraging this hybrid model, engineers can achieve more reliable and precise predictions, ultimately enhancing the safety and efficiency of infrastructure projects.

### Statistical results

To further assess the performance of the proposed models, a thorough analysis was carried out using various statistical measures, and the results are listed in Table 6. By scrutinizing the performance metrics across all datasets, it is evident that the hybrid ANN-BBO model has significantly improved in reducing errors and, as a result, increasing accuracy compared to the single ANN model. More specifically, the error-related metrics—MAE, RMSE, PI, and OBJ—demonstrate notable reductions in error by 20%, 56%, 57%, and 57%, respectively, when comparing the hybrid model’s (ANN-BBO) performance to the single model (ANN) on all compressive strength data. Likewise, a comparable trend is experienced in the errors of the predicted STS data. Besides, statistical metrics such as NSE, VAF, and A10-index indicate a strong goodness-of-fit and high efficiency of the ANN-BBO model. For example, regarding the A10-index, the ANN-BBO model achieves A10-index values of 0.988 for CS and 1 for STS across all datasets, while the corresponding values in the single ANN model are 0.929 and 0.940, respectively. Consequently, the statistical results indicate that the performance of the ANN-BBO model could surpass what the single ANN model offers, highlighting the performance improvement of the proposed hybrid model.

**Table 6:** Comparing the evaluation criteria.

Model	Feature	Data set	Metrics							
			R^2^	MAE (MPa)	RMSE (MPa)	NSE	PI	VAF (%)	OBJ (MPa)	A10-index
ANN	Compressive strength	Train	0.9424	0.024	2.137	0.941	0.028	94.169	0.781	0.941
		Validate	0.9563	0.026	1.836	0.955	0.025	95.535	0.142	0.920
		Test	0.9463	0.029	2.368	0.931	0.031	94.459	0.183	0.880
		All	0.9412	0.025	2.157	0.940	0.028	94.049	1.124	0.929
	Splitting-tensile strength	Train	0.9150	0.045	0.214	0.911	0.028	91.230	0.095	0.924
		Validate	0.9334	0.045	0.190	0.932	0.025	93.330	0.018	1.000
		Test	0.9246	0.043	0.222	0.905	0.029	90.869	0.020	0.960
		All	0.9174	0.045	0.212	0.914	0.028	91.411	0.134	0.940
ANN-BBO	Compressive strength	Train	0.9864	0.022	1.028	0.986	0.013	98.641	0.371	0.983
		Validate	0.9925	0.019	0.787	0.992	0.011	99.247	0.060	1.000
		Test	0.9969	0.014	0.591	0.996	0.008	99.625	0.045	1.000
		All	0.9887	0.020	0.942	0.989	0.012	98.872	0.484	0.988
	Splitting-tensile strength	Train	0.9840	0.019	0.091	0.984	0.012	98.395	0.039	1.000
		Validate	0.9814	0.022	0.109	0.978	0.014	98.118	0.010	1.000
		Test	0.9758	0.025	0.113	0.975	0.015	97.573	0.010	1.000
		All	0.9819	0.020	0.097	0.982	0.012	98.185	0.059	1.000

Statistical results reveal that the model exhibits a high degree of accuracy across various metrics, demonstrating its effectiveness in predicting compressive and splitting-tensile strengths with minimal error. These findings highlight the robustness of the ANN when combined with the biogeography-based optimization (BBO) algorithm, positioning this hybrid approach as a reliable tool for structural strength prediction in engineering applications. The model’s superior performance underscores its potential to enhance the precision and efficiency of material strength assessments in civil engineering.

### Role of input parameters in modeling

To assess the contribution of each input parameter in modeling the CS and STS of RCC, a relevancy factor (RF) analysis is conducted. The absolute RF value for each parameter reflects its extent of importance in determining the strength properties of the RCC, while its sign indicates whether its effect is positive or negative on the model’s outcome. The RF equation is defined as follows:

7 $${RF}_{({X}_{i }) }=\frac{\sum_{N}{(X}_{i,N}- \overline{{X }_{i}})({Y}_{N,p}-\overline{{Y }_{p}})}{\sqrt{{\sum_{N}{(X}_{i,N}- \overline{{X }_{i}})}^{2}{\sum_{N}{(Y}_{N,p}- \overline{{Y }_{p}})}^{2}}}$$

where *X*_*i*_: *i*th input parameter (*i* = 1, …, 12, based on the number of input parameters defined), *X*_*i,N*_: Value of *X*_*i*_ for the *N*th data point, $\overline{{X }_{i}}$: Average of the values of the input parameter with index *i*, *Y*_*N,p*_: Predicted value of the *N*th data point, $\overline{{Y }_{p}}$: Average of the values predicted.

**Fig. 11:**
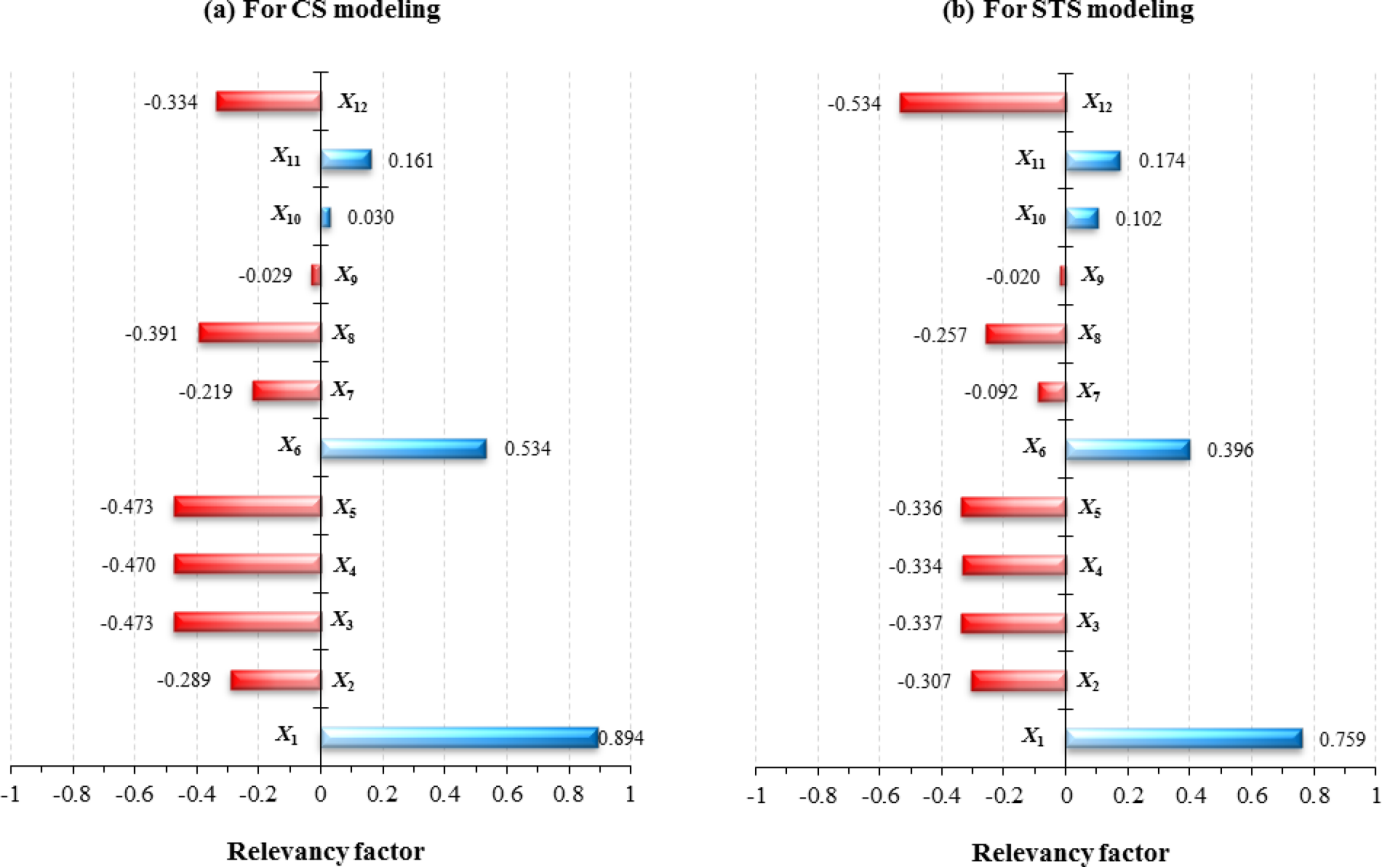
RF values of each input parameter (*X*_1_–*X*_12_) in modeling (**a**) CS and (**b**) STS using the best-proposed model.

**Fig. 12:**
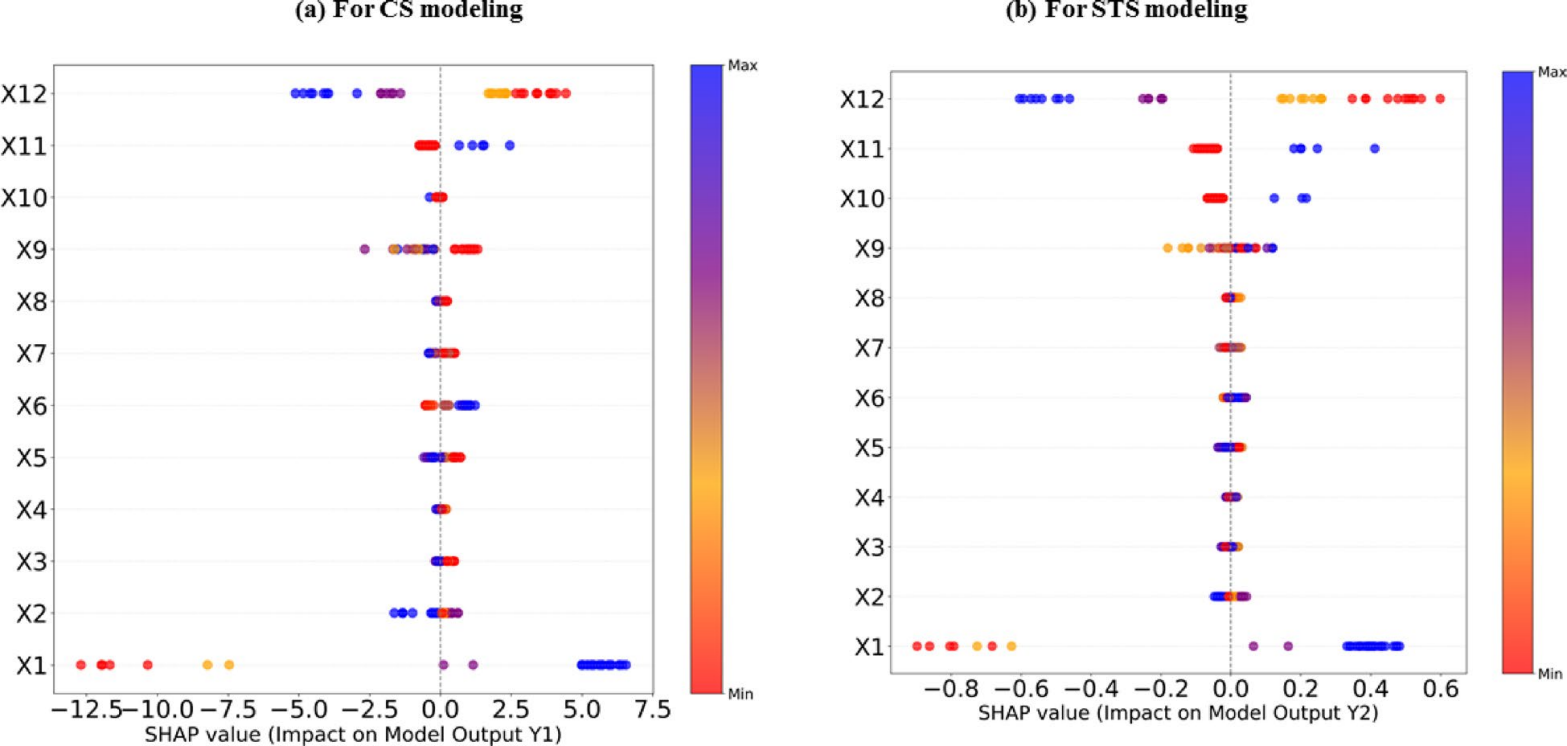
Results of SHAP analysis for (**a**) CS and (**b**) STS using the best-proposed model.

Figure 11 displays the results of this analysis for both CS and STS using the outputs obtained from the best-proposed model (ANN-BBO). Both outputs are regarded as strength properties, so the influence of the parameters follows the same trend, though there are slight differences in the values. As shown in Fig. 11, parameters *X*_1_ (cement) and *X*_6_ (water) have the most significant positive effect on the strength prediction results, which is logical given the well-known prominent role of these two components on the strength properties of RCCs^56^. On the other hand, parameter *X*_9_ (set retardant admixture) is identified as the parameter with the least impact on the strength properties. Among them, for CS performance, the parameters related to aggregate size (i.e., *X*_3_–*X*_5_) exhibit a strong inverse relationship, while for STS performance, the most negative effect is recorded by parameter *X*_12_ (waiting time after produce a layer). To further evaluate the importance of input parameters, SHapley Additive exPlanations (SHAP), a game theory-based method, is employed to interpret the contribution of each input parameter to the model’s output^57^. Figure 12 illustrates the significance of each feature by showing its impact on prediction accuracy. The color gradient represents the value of each feature, ranging from low (red) to high (blue). The horizontal axis of the plot indicates each feature’s effect on the model predictions, showing whether the influence is positive or negative. A positive impact implies that the feature increases prediction accuracy for the given sample, while a negative impact suggests a decrease in accuracy. A comparison of the RF and SHAP analyses clearly shows that both yield the same results. Similarly, in SHAP analysis for both CS and STS, parameter *X*_1_ (cement) is identified as the most important, and the repeated presence of data in the blue region indicates its positive effect. In contrast, parameter *X*_12_ shows a negative impact on both outputs. Consequently, both analyses show similar results for the contribution of each input parameter in modeling the CS and STS of RCC. These analyses are valuable as they elucidate how each input parameter influences the model’s output, offering a deeper understanding of the parameters’ roles and their impact on the model’s predictions.

### Future works

Future research should prioritize refining the identified parameters to enhance both CS and STS while exploring alternative materials or innovative mixing methodologies that may yield superior performance. Additionally, investigating the complex interactions among these parameters could reveal synergistic effects that not only improve material properties but also optimize production processes. Furthermore, integrating AI techniques to analyze these interactions could provide valuable predictive insights, enabling researchers to fine-tune material compositions with greater precision for specific applications.

Implementing real-time strength prediction with ANN and IoT sensors can help monitor compressive and splitting tensile strength, preventing substandard batches. Sensor-based mix adjustment using moisture sensors and PLC feedback algorithms may help adjust water content for consistent workability. An AI-based mix design optimizer, leveraging ANN and Biogeography-Based Optimization (BBO), could balance performance, cost, and sustainability. Batch scheduling assistants, based on decision rules and environmental data, are recommended to optimize production timing and reduce delays. Finally, adopting a digital twin system with IoT, BIM, and AI simulations can offer real-time monitoring and holistic optimization of the concrete production process.

## Conclusions

The compressive strength and splitting tensile strength of roller compacted concrete were statistically modeled using data obtained from an experimental study aimed at preventing cold joint formation.

In conclusion, the ANN model predicted CS values with R^2^ values of 0.9424, 0.9563, and 0.9463 for the training, validation, and test datasets, respectively, while the ANN-BBO model demonstrated superior performance with R^2^ values of 0.9864, 0.9925, and 0.9969, indicating a stronger correlation and more precise alignment with the ideal regression line (y = x). The CS modeling results reveal that over 90% of the ANN-BBO model’s prediction errors fall within [− 2 MPa, 2 MPa], whereas the ANN model exhibits a wider error range of [− 4 MPa, 4 MPa]; a similar trend is observed for STS predictions, where the ANN-BBO model confines more than 90% of values within [− 0.2 MPa, 0.2 MPa], outperforming the ANN model, which shows greater deviations, thereby confirming the ANN-BBO model’s superior reliability in predicting RCC strength properties.

Performance metric analysis across all datasets further highlights the ANN-BBO model’s enhanced predictive accuracy and error reduction, with MAE, RMSE, PI, and OBJ showing reductions of 20%, 56%, 57%, and 57%, respectively, in compressive strength data, alongside a similar trend in STS predictions, while statistical indicators such as NSE, VAF, and A10-index further affirm the model’s strong fit and efficiency. Ultimately, the statistical findings validate that the ANN-BBO model significantly surpasses the standalone ANN model, emphasizing the substantial performance improvements achieved through the proposed hybrid approach, which not only enhances accuracy but also delivers more reliable predictions, solidifying its effectiveness in comparison to traditional methods.

## Data Availability

The dataset that supports the findings of this study is not publicly available due to contractual limitations. However, key summary data and analyses are presented within the article. Further details may be available from the corresponding author upon reasonable request.

## Abbreviations

AI: Artificial intelligence
ANFIS: Adaptive neuro-fuzzy inference system
ANN: Artificial neural network
ANOVA: Analysis of variance
BBO: Biogeography-based optimization
BGG: Bagging algorithms
BR: Bayesian regularisation
CHAID: Chi-square automatic interaction detection
CNN: Convolutional neural networks
CS: Compressive strength
DSS: Decision support system
ELM: Extreme learning machine
FL: Fuzzy logic
GA: Genetic algorithm
GB: Gradient boosting
GBM: Gradient boosting machine
GEP: Gene expression programming
GOA: Grasshopper optimisation algorithm
HIS: Habitat suitability index
HVFA: High volume fly ash
LM: Levenberg–Marquardt
M5p: M5prime
M5r: M5rule
MAE: Mean absolute error
MARS: Multivariate adaptive regression splines
ML: Machine learning or multiple linear regression
MRA: Multiple regression analysis
NSE: Nash–Sutcliffe efficiency
OBJ: Objective function
PI: Performance index
PSO: Particle swarm optimization
RAP: Recycled asphalt pavement
RCC: Roller compacted concrete
RF: Random forest
RMSE: Root mean squared error
SCG: Scaled-conjugate gradient
SHAP: SHapley Additive exPlanations
SHM: Structural health monitoring
SIVs: Suitability index variables
STS: Splitting-tensile strength
SVR: Support vector machines
VAF: Variance accounted for
